# A forty-year review of Rocky Mountain spotted fever cases in California shows clinical and epidemiologic changes

**DOI:** 10.1371/journal.pntd.0010738

**Published:** 2022-09-15

**Authors:** Anne M. Kjemtrup, Kerry Padgett, Christopher D. Paddock, Sharon Messenger, Jill K. Hacker, Tina Feiszli, Michael Melgar, Marco E. Metzger, Renjie Hu, Vicki L. Kramer

**Affiliations:** 1 California Department of Public Health, Sacramento, California, United States of America; 2 California Department of Public Health, Richmond, California, United States of America; 3 Centers for Disease Control and Prevention, Atlanta, Georgia, United States of America; 4 School of Medicine, University of California, San Francisco, California, United States of America; 5 California Department of Public Health, Ontario, California, United States of America; Mahidol Oxford Tropical Medicine Research Unit, THAILAND

## Abstract

Rocky Mountain spotted fever (RMSF) is a life-threatening tick-borne disease documented in North, Central, and South America. In California, RMSF is rare; nonetheless, recent fatal cases highlight ecological cycles of the two genera of ticks, *Dermacentor* and *Rhipicephalus*, known to transmit the disease. These ticks occur in completely different habitats (sylvatic and peridomestic, respectively) resulting in different exposure risks for humans. This study summarizes the demographic, exposure, and clinical aspects associated with the last 40 years of reported RMSF cases to the California Department of Public Health (CDPH). Seventy-eight RMSF cases with onsets from 1980 to 2019 were reviewed. The incidence of RMSF has risen in the last 20 years from 0.04 cases per million to 0.07 cases per million (a two-fold increase in reports), though the percentage of cases that were confirmed dropped significantly from 72% to 25% of all reported cases. Notably, Hispanic/Latino populations saw the greatest rise in incidence. Cases of RMSF in California result from autochthonous and out-of-state exposures. During the last 20 years, more cases reported exposure in Southern California or Mexico than in the previous 20 years. The driver of these epidemiologic changes is likely the establishment and expansion of *Rhipicephalus sanguineus* sensu lato ticks in Southern California and on-going outbreaks of RMSF in northern Mexico. Analysis of available electronically reported clinical data from 2011 to 2019 showed that 57% of reported cases presented with serious illness requiring hospitalization with a 7% mortality. The difficulty in recognizing RMSF is due to a non-specific clinical presentation; however, querying patients on the potential of tick exposure in both sylvatic and peridomestic environments may facilitate appropriate testing and treatment.

## Author summary

Our analysis of the last 40 years of reported Rocky Mountain spotted fever (RMSF) in California included cases that met specific inclusion criteria which highlighted new information. First, there are two distinct epidemiologic patterns which are illustrated with detailed descriptions of two fatal cases. The typical sylvatic cycle involves transmission by *Dermacentor* spp. ticks in grassland or woodland habitats. A peridomestic cycle exists that involves transmission by the brown dog tick, *Rh*. *sanguineus*, often in association with travel to hyper-endemic areas such as certain parts of northern Mexico or resulting from the increasing presence of *Rh*. *sanguineus* in Southern California. Second, the typical clinical picture of fever, headache, chills, and rash emphasized the non-specific nature of this life-threatening disease. Inquiries regarding recent tick exposure in both the sylvatic and peridomestic environments can help in recognition of the disease. Lastly, while RMSF remains rare in California, incidence is increasing, notably in the Hispanic/Latino population. Public health messaging about RMSF in California must focus on both sylvatic and peridomestic settings, particularly in Southern California, and should be culturally appropriate.

## Introduction

*Rickettsia rickettsii*, a tick-borne species of the spotted fever group *Rickettsia* (SFGR), causes Rocky Mountain spotted fever (RMSF), and occurs in North, Central and South America [1]. As the most pathogenic spotted fever rickettsiosis, RMSF can progress rapidly from a febrile illness to life-threatening disease with hemorrhagic complications [2]. If untreated with tetracycline-class antibiotics [3], doxycycline being the drug of choice [4], RMSF can result in death. The case-fatality of this disease in the pre-antibiotic era was 23% [5, 6]. Since initial clinical signs are nonspecific, disease recognition and diagnosis can be difficult, potentially resulting in fatal outcomes [5, 7–9].

In the United States [US), RMSF has been nationally reportable since the 1920s with multiple case definition changes over time as epidemiologic and diagnostic understanding of the disease has evolved [10]. In the US, reports of RMSF are increasing [4, 11], principally driven by the reporting of cases fitting the national surveillance criteria for a probable (as opposed to confirmed) case [11, 12]. For example, ELISA tests and commercial laboratory positive cut-off values were included in the 2010 case definition for probable cases, resulting in more positive laboratory tests being reported [11]. In addition, recognition of other spotted fever group *Rickettsia* [SFGR) that cross react with *R*. *rickettsii* antigens (e.g., *R*. *parkeri*, *R*. *akari*, and *R*. 364D*)* [13, 14] prompted the 2010 case definition change that placed RMSF reporting under Spotted Fever Rickettsioses (which included aforementioned *Rickettsia* species). The potential for false positive IgM tests [15] and nonspecific IgG titers ≤ 1:64 [16] resulted in changes to the SFGR case definition in 2020, when IgM titers as laboratory support was excluded and increasing the IgG positive cut-off reciprocal titer to 128 improved the specificity of the case definition. *Rickettsia typhi*, transmitted by fleas, is also endemic in areas of Southern California [17], and may serologically cross-react with SFG tests. Estimates for serologic test performance suggest that SFG tests have slightly better sensitivity and specificity than tests for *R*. *typhi* and differentiation relies on exposure history and potentially stronger reactivity to the specific antigen if both agents suspected.[18]. In California, RMSF cases have been documented since the early 20^th^ century [19]. From 1980 to 2019, 0 to 3 confirmed and 0 to 14 probable cases (average of approximately one each annually) were reported [20].

Known tick-vector species of RMSF in the US include the Rocky Mountain wood tick (*Dermacentor andersoni*) [21] and the American dog tick (*D*. *variabilis)* [21, 22]. *Dermacentor andersoni* occurs in the Sierra Nevada mountains and in the far northeastern counties of California [23]. Western populations of ticks formerly classified as *D*. *variabilis* are now considered a separate species that is designated as *D*. *similis* [24] which occur in California, in wooded foothill areas as well as in lower elevation areas and dryer habitats. [25–27]. *Rickettsia rickettsii* has never been detected in either of these species in California [28, 29]. In contrast, *R*. *rickettsii* has been molecularly detected in the Pacific Coast tick (*D*. *occidentalis*) [30]. *Dermacentor occidentalis* ticks are typically found in wildland areas of California particularly in moist coastal or wooded foothill areas [25] and can overlap with the distribution of *D*. *similis*.

The brown dog tick (*Rhipicephalus sanguineus*) has been identified as an effective RMSF vector in the US [31]. A large urban outbreak of RMSF has persisted in Mexicali, the capital of Baja California, since 2009 [32–34]. In addition, molecular detection of *R*. *rickettsii -*infected *Rh*. *sanguineus* ticks in Southern California [35] highlights this species as a peridomestic vector for RMSF in this region. Several studies support the premise of *Rh*. *sanguineus* s.l. as an important vector of RMSF along the US-Mexico border. Drexler et al. [36] described four fatal RMSF cases along the US-Mexico border where *R*. *rickettsii* was molecularly detected from one brown dog tick from a California case-patient’s home. Estrada, et al. [37] found that *Rickettsia* spp. seroprevalence among dogs increased with closer proximity to the border, and Lopez-Perez et al. [38] described DNA detection of *R*. *rickettsii* from coyotes and *Rh*. *sanguineus* s.l. at the California-Mexico border. Human exposure to *Rh*. *sanguineus* s.l. is primarily associated with exposure to domestic dogs in peridomestic and rural settings [39].

The intent of this study was to include and describe the epidemiology of human cases that most likely are RMSF through conservative case inclusion, excluding other zoonotic SFGR that have slightly different eco-epidemiology such as *Rickettsia* 364D [40]. The objectives are to: 1) present two fatal human cases of RMSF in California which illustrate exposure to two different RMSF tick vectors, 2) contextualize these fatal cases with an epidemiological summary of RMSF human cases reported to the California Department of Public Health (CDPH) from 1980 to 2019, and 3) provide a more detailed clinical summary of cases from 2011 to 2019, afforded by electronic surveillance, to emphasize the clinical challenge of recognizing RMSF. The epidemiology of RMSF in California described in this study can inform public health preventive messaging and guide response, while highlighting the need for increased medical awareness for this potentially fatal disease.

### Case 1: (Fresno County, *Dermacentor* exposure)

In December 2012, a 35-year-old male, previously in good health, presented to an emergency department in Fresno County, California, complaining of four-day history of headache, nausea, vomiting, and diarrhea. At admission, the patient had a fever of 39.3°C (102.7°F) and was hypotensive (80/67mmHg). A nodule was noted on the anterior side of his elbow which the patient said was a result of an insect bite. Major laboratory abnormalities included elevated creatinine (2.0 mg/dL), elevated AST (55 U/L), slight hyponatremia (132 mEq/L), lymphopenia (5mm^3^), and neutropenia (30/mm^3^) with increased immature leukocytes (bands) (52 mm^3^). Due to increasing severity of symptoms, the patient was hospitalized. Two days after hospitalization, a mottled rash developed on his lower abdomen and lower extremities, accompanied by altered mental status. Differential diagnoses were general and included pneumonia, pyelonephritis, viral syndrome and sepsis, with no etiology noted. The patient was transferred to the ICU where, despite fluid and vasopressor support and broad-spectrum antibiotics (ceftriaxone, vancomycin), the patient suffered cardiac arrest and died three days post admission.

Consultation with state public health officials prompted collection at autopsy of post-mortem serum and tissue of lung, liver, spleen, kidney, heart, and cerebral cortex to test for hantavirus, rabies, *Leptospira* spp., and SFGR. Serology and molecular tests performed at the CDPH Viral and Rickettsial Disease Laboratory (VRDL) were serologically negative (both IgG and IgM) and molecular tests were similarly negative for all disease agents. Molecular and immunohistochemistry testing of tissues was also performed at the US Centers for Disease Control and Prevention (CDC). All tissues were positive by immunohistochemical staining for SFGR and were negative for the other aforementioned agents. Kidney tissue was reported as PCR positive (ompA and ompB genes) for SFGR with the ompA product noted as sequence-positive for *Rickettsia rickettsii* (sequence not published as this was from a diagnostic specimen).

History obtained from the patient’s family revealed that as an avid bird hunter, he traveled weekly to a local hunting area near his home in the California Central Valley with dogs, including 10 days prior to disease onset.

Follow-up at the patient’s home and hunting localities by the CDPH Vector-Borne Disease Section (VBDS) found no evidence or likely habitat for ticks around the home, and the patient’s dog was free of ticks, though a family member noted that one dog acquired several ticks after a November hunting date. In the area where the patient hunted, four *D*. *occidentalis* and 44 *D*. *variabilis* ticks were recovered from late April through mid-May 2013, about five months post-exposure. Ticks were tested by PCR for *R*. *rickettsii* as previously described [30] and all tested negative.

### Case 2: (Riverside County, *Rhipicephalus* exposure)

In May 2014, a 52-year-old female from Calexico, California presented to a local emergency department following three days of fever, diarrhea, nausea, and vomiting. A clinical description of this case has been published elsewhere [36] with some additional details provided herein. The patient was diagnosed with presumed urosepsis and transferred to a tertiary care facility. A serum sample from day 24 of illness demonstrated reciprocal IgG titer of 256 to *R*. *rickettsii*. The patient was intubated and put on hemodialysis due to acute renal failure. Despite treatment with broad spectrum antibiotics including doxycycline beginning day 26 post onset, she developed disseminated intravascular coagulation and died two days later. Retrospective testing of sera from days 7 and 26 of illness demonstrated four-fold increase in reciprocal IgG titer to *R*. *rickettsii* from <64 to >1,024 at CDPH-VRDL. Previously described autopsy results [36] showed widespread ischemic damage through multiple organs; a post-mortem skin biopsy was PCR positive for SFGR using a SYBR Green quantitative rOmp-A gene PCR assay [41], providing further evidence supporting this case as a SFGR case, most likely RMSF. The patient had no travel history within one month prior to onset, though family members travelled frequently across the border to Mexicali, Mexico, bringing their pet dog with them. Follow-up by CDPH-VBDS identified a *Rh*. *sanguineus* s.l. infestation of the patient’s dogs and yard. Of 37 ticks collected and molecularly tested and sequenced, one (2.7%) was positive for *R*. *rickettsii* [36]. A local health department alert was issued to increase physician awareness and local vector control agencies informed local veterinarians and residents with material in English and Spanish regarding potential disease risk associated with brown dog tick infestations.

## Methods

### Ethics statement

The Committee for the Protection of Human Subjects at the California Health and Human Services Agency determined that this project number 2021–059 is exempt under their criteria for review. This decision was issued under the California Health and Human Services Agency’s Federal-wide Assurance #00000681 with the Office of Human Research Protections.

Previously classified cases of RMSF reported to CDPH from 1980 to 2019 were reviewed. The national RMSF case definition changed over the study period. To standardize case inclusion for this study, we defined a “confirmed” and “probable” case based on the following definition, with recognition that these classifications represent a surveillance definition, not medical definition. A confirmed RMSF case was defined as a case-patient reporting compatible illness (measured or reported fever plus one of the following: chills, sweats, headache muscle pain, joint pain, eye pain, gastrointestinal involvement, rash, cough, or hypotension) and confirmatory laboratory results of a four-fold titer change (complement fixation or immunofluorescent antibody [IFA] test [IgG]), DNA detection, or positive immunohistochemical antibody [IHA] staining of tissue). A probable RMSF case was defined as a person reporting compatible illness with supportive laboratory results of an elevated IgG IFA titer ≥ 1:128. *Rickettsia* 364D, *R*. *parkeri* and *R*. *akari*, SFGR emerging in California and parts of the US [4, 40], present with an eschar with serologic cross reactivity to RMSF antigen. Because the eco-epidemiology of these eschar-producing mild SFGR is different than *R*. *rickettsii* [14, 40], and though eschars have rarely been described in RMSF cases [42] to focus this review on RMSF, cases reporting an eschar only as the clinical symptom were not included. Similarly, reported case-patients who lived in California counties endemic for *R*. *tyhphi* (Los Angeles and Orange) were included if exposure history did not suggest local *R*. *typhi* exposure or if tested for *R*. *typhi*, had four-fold higher titer of SFGR than *R*. *typhi* titer.

Data were de-identified, and based on CDPH data policy guidelines, available and analyzed behind the CDPH firewall. Authors responsible for the data analysis had access to the data as described, as did all other CDPH collaborators should they have needed it; non-CDPH collaborators did not require the information for collaboration. Aggregate data reflective or our dataset are publicly available via CDPH Surveillance and Statistics data reports and epidemiologic summaries: https://www.cdph.ca.gov/Programs/CID/DCDC/Pages/SSS.aspx. Data requests may be submitted to the CDPH- Surveillance and Statistics Section, 916-552-9720, or email: IDB-SSS@cdph.ca.gov. Data used to make charts are available in S1 Data.

Analysis of cases over time were performed by grouping cases in 20-year blocks (1980 to 1999 and 2000 to 2019) to have adequate case numbers in comparable time periods. Reported symptoms were analyzed from cases beginning in 2011 when a standardized electronic case report form became available via the California Reportable Disease Information Exchange (CalREDIE) [43], facilitating symptom reporting.

Statistical analysis was performed using Epi-Info (Centers for Disease Control and Prevention, Atlanta, GA) and Excel (Microsoft Corporation, Redmond, WA). Statistical analyses were univariate comparisons using Z-tests for proportions, t-tests to compare means, χ^2^ test with two-tailed p for two-by-two comparisons, and an incidence rate ratio with 95% confidence intervals to compare incident rates [44] by ethnicity over time. P-values less than 0.05 were considered significant; 95% confidence intervals for the incidence rate ratio were used to evaluate significance. For calculating incidence and the incidence rate ratio, population estimates for Hispanic/Latino and non-Hispanic/non-Latino in appropriate time periods were obtained from the California Department of Finance Demographic Research Unit [45]. California was divided into northern and southern regions for comparison; the Southern California region was comprised of Imperial, Kern, Los Angeles, Orange, Riverside, San Bernardino, San Luis Obispo, Santa Barbara, San Diego and Ventura Counties, and the northern region was comprised of the remaining 48 counties.

## Results

**Fig 1:**
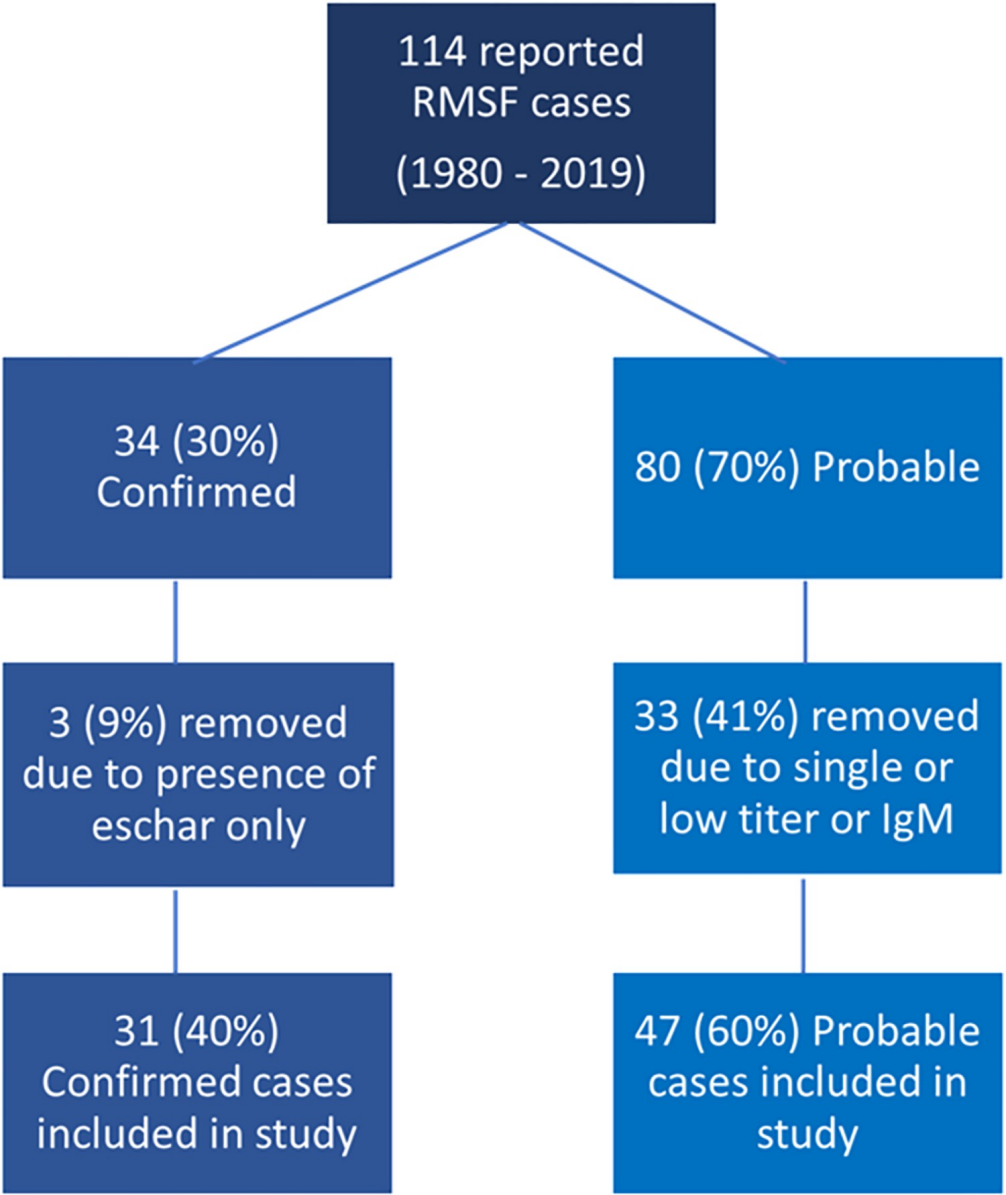
Flow of case evaluation for inclusion in this analysis. Case reports retrieved from 1980 to 2019 include all those reported as Rocky Mountain spotted fever (RMSF). Conservative case inclusion criteria were applied as described in methods to focus on those cases most likely to be RMSF. Included in the analysis were 31 confirmed cases and 47 probable cases.

Fig 1 details the case acquisition and evaluation flow.

From 1980 to 2019, 114 cases of RMSF were reported to CDPH. Following national case definitions at time of reporting, 34 (30%) were considered confirmed and 80 (70%) were considered probable. After applying the inclusion criteria for this study, three (9%) of the confirmed cases were not included due to presence of an eschar and 33 (41%) of the probable cases were not included due to a single or repeated low titer (≤1:64) or IgM-only testing.

**Fig 2:**
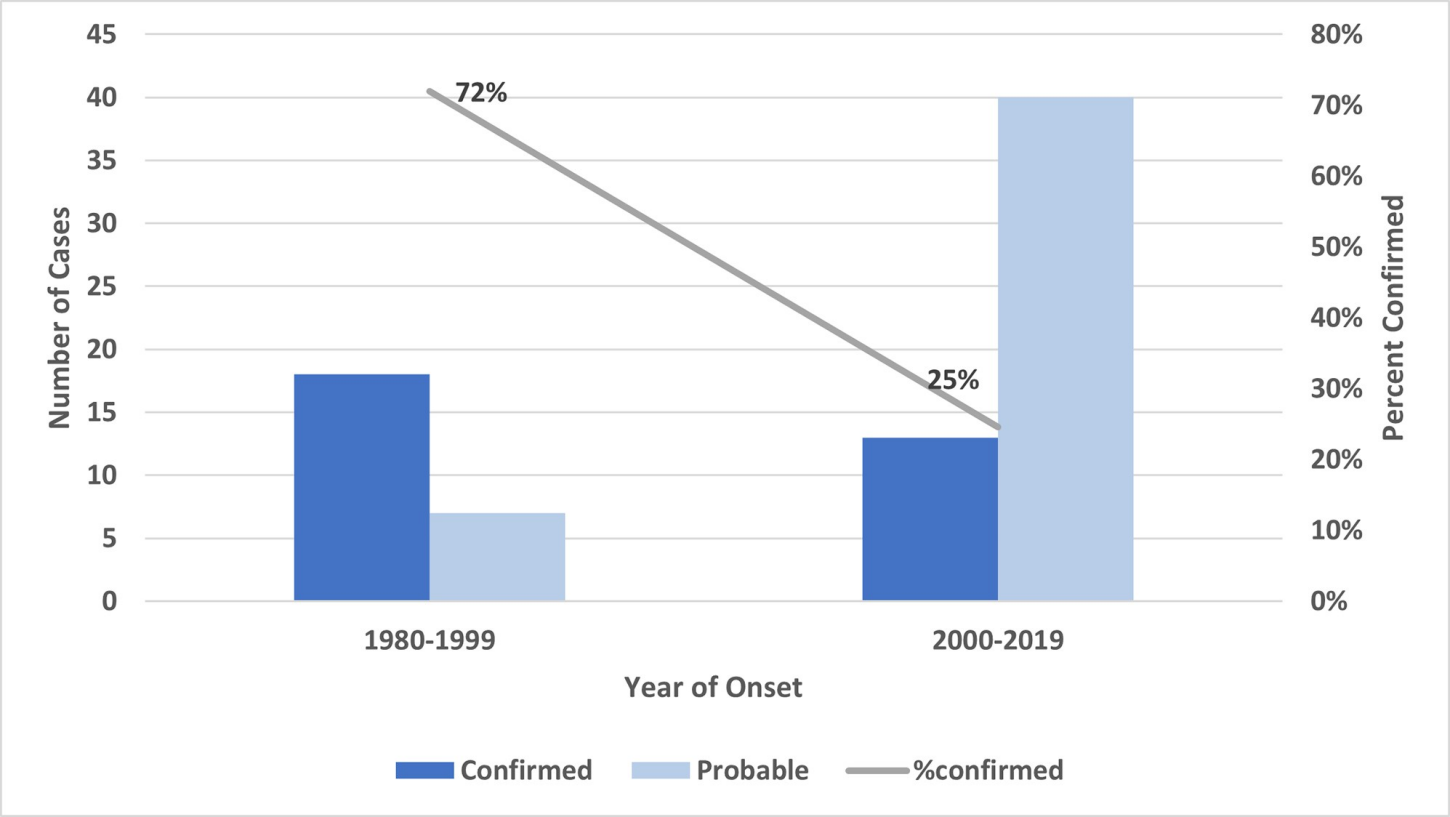
Confirmed and probable cases of RMSF in California and percent of reported cases confirmed in two 20-year time blocks, 1980 to 1999 and 2000 to 2019.

Of the 78 resultant cases included in this study, 31 (40%) were confirmed and 47 (60%) were probable. Three (4%) of the cases were fatal; these were confirmed cases occurring in 2012 (Case 1 above) and 2014 (2 cases, including Case 2 above). Total reported cases increased from 25 during 1980–1999 to 53 during 2000–2019 with the percentage of confirmed cases significantly decreasing from 72% of reported cases to 25% of reported cases (p<0.05). (Fig 2).

Table 1 compares demographics, hospitalization, and fatality of cases in the two reporting blocks, 1980 to 1999 and 2000 to 2019. Forty-nine (63%) cases were male; 29 (37%) were female; median age was 44 years (interquartile range 32–61 years), and age did not differ by sex (t-test, p > 0.05). These demographic data were comparable between the two 20-year reporting blocks (1980 to 1999 and 2000 to 2019) (p >0.05).

**Table 1:** Demographics, hospitalization, and fatality of reported RMSF cases in California in two 20-year reporting blocks, 1980 to 1999 and 2000 to 2019. Asterisks indicate where there are statistically differences in proportions with a Z test, p < 0.05 considered significant.

Demographics, hospitalization, and fatality	1980–1999 n = 25	2000–2019 n = 53	Total (n = 78)
Sex			
Male (%)	18 (72)	31 (59)	49 (63)
Female (%)	7 (28)	22 (41)	29 (37)
Median age (range)	40 (4–79)	48 (5–81)	44 (4–81)
Race			
White (%)	21 (84)	31 (58)	52 (67)*
Black (%)	2 (8)	3 (6)	5 (6)
Asian (%)	0 (0)	2 (4)	2 (3)
Other/Unknown (%)	2 (8)	17 (32)	19 (24)*
Ethnicity			
Non-Hispanic/Non-Latino (%)	22 (88)	21 (40)	43 (55)*
Hispanic/Latino (%)	1 (4)	20 (38)	21 (27)*
Unknown (%)	2 (8)	12 (23)	14 (18)
Hospitalization			
Hospitalized (%)	15 (71)	30 (58)	45 (62)
Unknown (%)	4 (16)	1 (2)	5 (6)
Fatality (%)	0 (0)	3 (6)	3 (4)

For self-reported race, there was a significant decrease between the two reporting blocks in proportion of cases self-reporting as white, from 84% to 58%, while the other/unknown category significantly increased from 8% to 32%. The trend in self-identifying as Hispanic/Latino increased significantly from 4% in 1980 to 1999 to 38% in 2000 to 2019, with a significant decrease in case-patients self-reporting as non-Hispanic/non-Latino in that same time-period from 88% to 40%. Hospitalization proportions did not differ significantly between the reporting blocks: 62% of all reported case-patients were hospitalized. Fatalities (3) were only reported in 2000 to 2019.

Statewide incidence increased significantly from 0.04 cases per million during 1980 to 1999 to 0.07 cases per million during 2000 to 2019 (risk ratio [RR] 1.6, 95% confidence interval [CI]: 1.0–2.7). Most of the increase in risk was seen in the Hispanic/Latino population (RR 11.3, 95% CI: 0.77–167.28), though the increase was not statistically significant (95% confidence intervals include 1; Table 2).

**Table 2:** Incidence of RMSF cases per million persons by ethnicity, using ethnicity-specific population estimates, 1980–2019. The Incidence rate ratio compares the ethnicity specific incidence between 2000–2019 (numerator) and 1980–1999 (denominator) and is a measure of excess risk between the two reporting blocks.

Ethnicity	Incidence 1980–1999 (95% CI)	Incidence 2000–2019 (95% CI)	Incidence rate ratio (95% CI)
Hispanic/Latino	0.006 (0.00002–0.004)	0.07 (0.04–0.11)	11.3 (0.77–167.28)
NonHispanic/NonLatino/Other	0.06 (0.04–0.08)	0.07 (0.05 - .10)	1.3 (0.7–2.2)
Total	0.04 (0.03–0.06)	0.07 (0.05–0.09)	1.6 (1.0–2.7)

**Fig 3:**
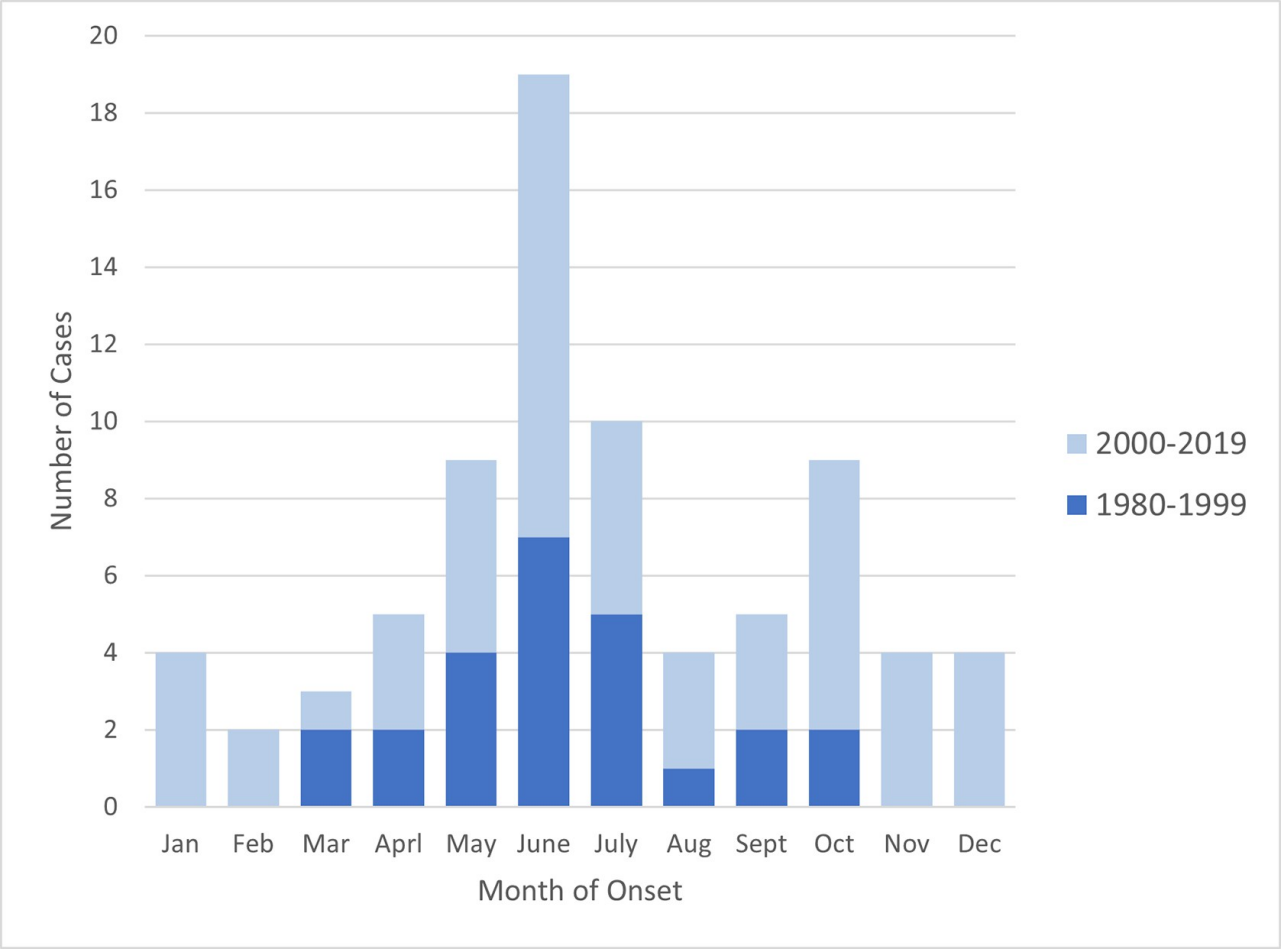
Number of RMSF cases (both confirmed and probable) by month of onset, 1980 to 2019, stratified by 20-year reporting blocks.

Onset month for cases showed a summer peak in June (Fig 3). The overall monthly pattern for onset was similar over time (p > 0.05), although onset of cases during 1980 to 1999 were only between March and October compared to year-round during 2000 to 2019. The month of onset did not differ for patients exposed in (n = 42) and out-of-state (n = 36). (p>0.05).

**Fig 4:**
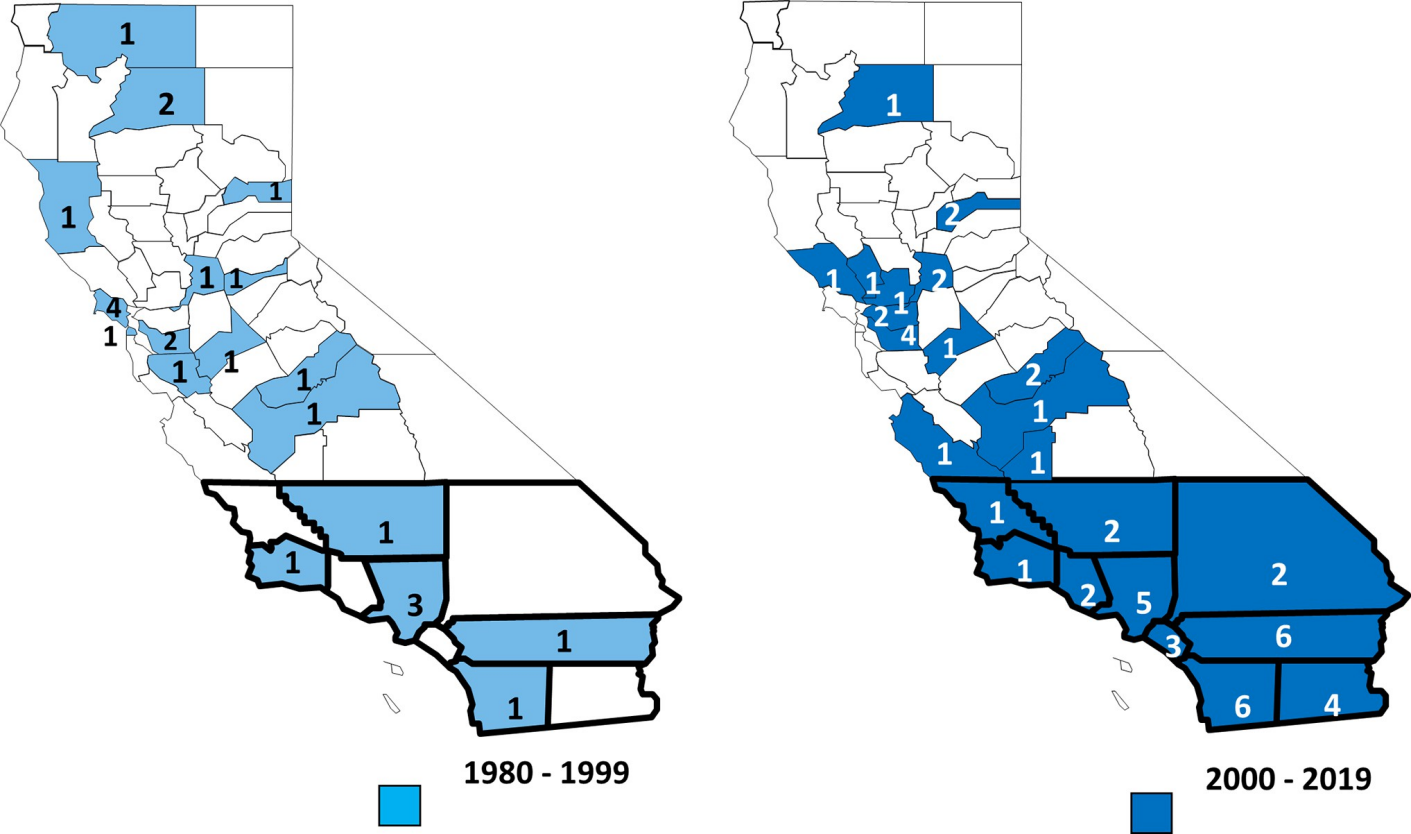
County of residence of RMSF cases, in 20-year reporting blocks, 1980–2019. Counties included in the southern region are heavily outlined. Source for base-layer map: File:California counties outline map.svg—Wikimedia Commons (https://commons.wikimedia.org/wiki/File:California_counties_outline_map.svg#filelinks).

Reported counties of residence of case-patients included 33 counties throughout California with Los Angeles reporting the highest number at 13 (16%) followed by Riverside and San Diego at 7 (8%) cases each. The number of cases reported from the southern region increased significantly (p = 0.004) from 8 (32%) of reported cases between 1980 and 1999 to 35 (66%) between 2000 and 2019 (Fig 4).

**Fig 5:**
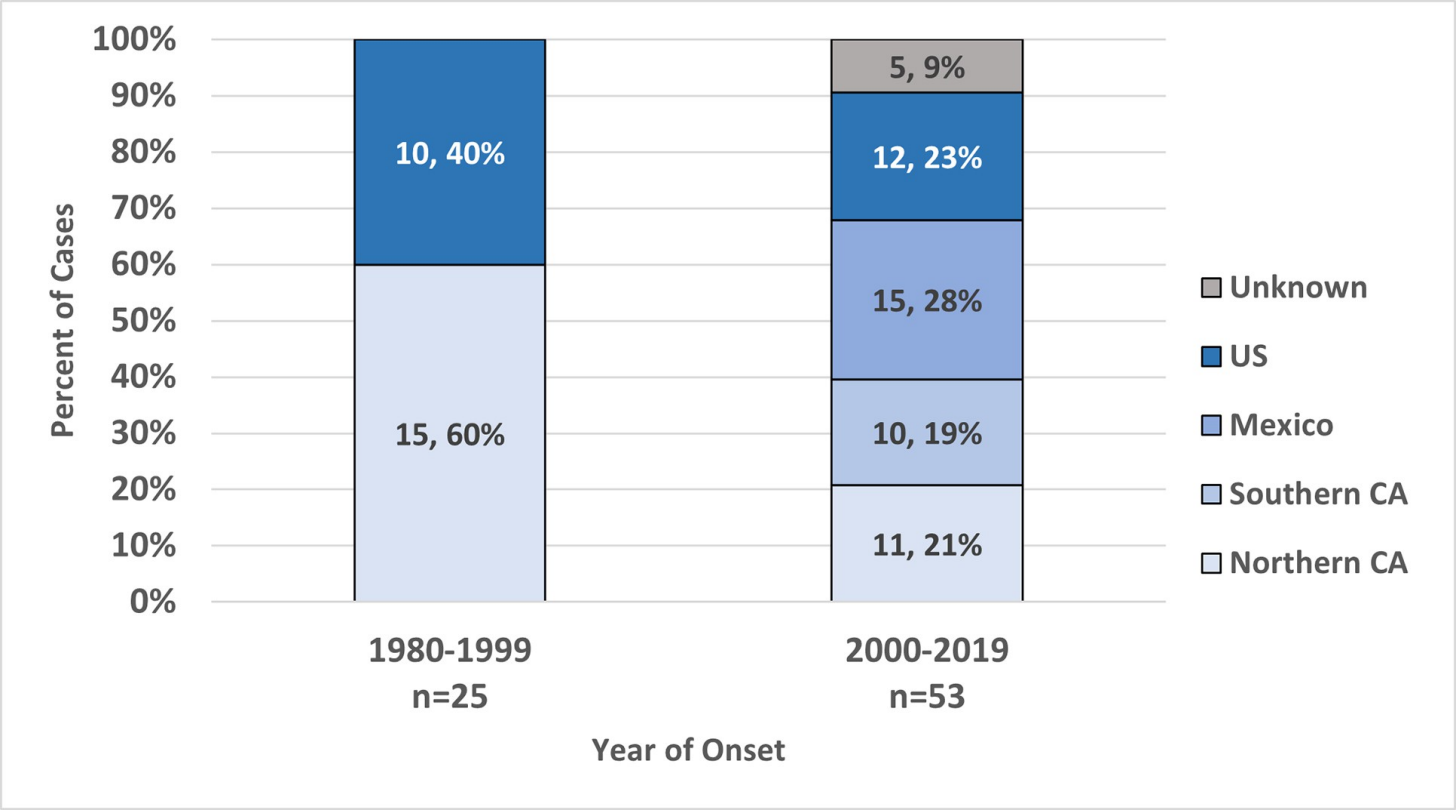
Number and percentage of California RMSF cases where regions of exposure prior to onset could be determined in 20-year reporting blocks, 1980–2019. Exposure regions are “Northern CA” (California counties north of San Luis Obispo, Kern and San Bernardino Counties; see Fig 4), “Southern CA” (counties not in Northern CA), “US” (any state outside California in the United States) and “Unknown” (travel not specified).

Exposure to RMSF was both local and travel associated. Of all cases, 48 (62%) reported travel outside of their county of residence 2 to 12 days prior to disease onset. Of these travel-associated cases, 23 (48%) reported travel to other parts of the US, 14 (29%) reported travel to Mexico, and 11 (23%) reported travel to other California counties. Considering areas of exposure for cases that both traveled and had no travel history, there was an increase in reported exposure to RMSF in Southern California counties and in Mexico in cases reported during 2000 to 2019 compared to those reported in previous years (p<0.01) (Fig 5).

Diagnosis of cases was based primarily on clinical signs and serological testing. Of the confirmed cases, a four-fold titer increase was seen in 27 (87%) cases (including Case 2 above), isolation of the *R*. *rickettsii* organism was possible in 2 cases (6%), a rash biopsy was positive by immunohistochemical staining in 1 (3%), and 1 (3%) confirmed case (Case 1 above) was serologically negative but post-mortem tissues were positive by PCR, sequencing, and immunohistochemical staining. (Case 2 above was also PCR positive in addition to serologic confirmation). All probable cases were diagnosed with compatible clinical signs and with titer values at their first, or in some cases second, blood draw ranging from 1:128 to 1:512 with 28 (60%) having at least one titer ≥1:256. There was no difference in median time from symptom onset date to the first serologic test between confirmed and probable cases (7 days, range 0 to 56 days for confirmed and 0 to 46 days for probable, p > 0.05). Among confirmed cases, median time between first and second sera samples was 21 days, range 8 to 30 days. Eight (22%) probable cases also had a second serologic test done that either did not demonstrate change in titer value between first and second titer, showed only a two-fold increase to 1:128, or had assays that were conducted by different laboratories so were not comparable. The median time between sample collection for the first and second titers among probable cases was slightly shorter (20.5 days) but not significantly different from confirmed cases (p >0.05).

When reported, exposure risk included insect bites, wildland activity, and animal exposure. Of the 32 (41%) cases reporting “insect bites,” 20 (62%) specifically described tick bites, whereas others indicated other potential insects. More confirmed cases (20, 65%) reported an insect bite compared to probable cases (12, 26%), p <0.001. A higher percentage of cases during 1980 to 1999 reported insect bites (17, 68%) than did cases during 2000 to 2019 (15, 28%) (p < 0.05) but there was no difference in percentage reporting a tick bite specifically (p >0.05). Of all cases, 19 (24%) described outdoor activity in wildland areas, and 9 (12%) described owning or direct contact with dogs and/or cats. There was no difference between confirmed and probable cases in terms of these latter two exposures.

Between 2011 and 2019, 9 confirmed and 37 probable cases were reported to CDPH through electronic disease reporting, facilitating consistent symptom data collection. Demographics and symptom summary are in Table 3; all characteristics were similar when comparing confirmed and probable cases (p > 0.05). Age distribution and sex ratios of 2011–2019 cases were also similar to cases prior to 2011 (p > 0.05).

**Table 3:** Case outcomes, signs, and symptoms from electronically reported RMSF cases, 2011–2019. There was no statistical difference in proportions between confirmed and probable cases for case outcomes, signs and symptoms (Z statistic for proportions, p >0.05.).

Case outcomes, signs, and symptoms	Confirmed (n = 9)	Probable (n = 37)	Total (n = 46)
Hospitalized (%)	7 (78)	19 (51)	26 (57)
Median number days hospitalized (range)	3 (2–13)	8.5 (1–24)	7 (1–24)
Fatalities (%)	3 (33)	0	3 (7)
Measured Fever * (%)	8 (89)	36 (97)	44 (96)
Headache (%)	7 (78)	27 (73)	34 (74)
Chills (%)	3 (33)	26 (70)	29 (63)
Rash (%)	7 (78)	21 (51)	28 (56)
Muscle pain (%)	2 (22)	19 (51)	21 (46)
Nausea/vomiting (%)	2 (22)	19 (51)	21 (46)
Abdominal Pain (%)	4 (44)	17 (46)	21 (46)
Sweats (%)	2 (22)	18 (49)	20 (43)
Joint pain (%)	1 (11)	18 (49)	19 (41)
Diarrhea (%)	5 (56)	10 (27)	15 (33)
Cough (%)	1 (11)	13 (35)	14 (30)
Hypotension (%)	4 (44)	8 (22)	12 (26)
Eye pain (%)	0 (0)	8 (22)	8 (17)
Other (%)	5 (56)	20 (54)	25 (54)

*Two cases reported subjective fever, not measured in clinical setting

The median number of symptoms reported was seven (range 1–13). Fever (96%), headache (74%), chills (63%), and rash (56%) were the most common symptoms reported by more than half the case-patients; 32 (70%) of cases reported three or more of these common symptoms. Rash descriptions included petechial, maculopapular, diffuse, or erythematous and on various body locations including arms, legs, abdomen, back, and occasionally on feet and hands. Included under “other” symptoms described by 25 (54%) of case-patients were neurologic-related symptoms (9–39%) such as altered mental status, weakness, lethargy, and photophobia; respiratory-related symptoms (4–17%) including congestion, shortness of breath, chest pain and respiratory failure; integumentary-related symptoms (3–13%) including skin sloughing, swollen wrists and ankles, and oropharyngeal ulcers; and sepsis and kidney involvement in 4 (17%) case-patients. Of the 31 (67%) reporting chemistry and hematology results, thrombocytopenia (14–45%), anemia (7–23%), leukopenia (6–19%), leukocytosis (4–13%), and elevated liver enzymes (3–12%) were the abnormalities reported; 10 (32%) reported values within normal limits. These values were similar between confirmed and probable cases (p > 0.05).

## Discussion

The two case descriptions are illustrative of an evolving picture of RMSF in California. Case-patients’ exposure reports suggested involvement of two different tick species. Careful site investigation of Case 1 suggested *Dermacentor* spp. tick exposure, as described historically in California [28]. The mid-December onset date suggested tick exposure in early December. While no specific records exist for *D*. *occidentalis* and *D*. *similis* collections in the suspected exposure location in December, both adult and immature life stages of both *Dermacentor* species have been collected in November and December in nearby California Central Valley counties [27, 40]. Detection of *R*. *rickettsii* DNA in *Dermacentor* ticks in California is rare and despite extensive surveillance, to date the only detection has been in *D*. *occidentalis* ticks in Riverside County [30]. The “typical” RMSF clinical picture of fever, headache, chills, and rash was also present in this case-patient; however, these nonspecific symptoms make diagnosis a challenge. Moreover, the negative RMSF serology underscores the importance of inquiring about tick exposure in order to include RMSF as a differential diagnosis and begin appropriate treatment.

Case 2 reflects the shift in the epidemiologic picture of RMSF in California with an increase in the proportion of cases reported from southern jurisdictions, the increase in reported cases with travel associated to Southern California counties and Mexico, and the increase in self-reported Hispanic/Latino ethnicity. The change in self-reported race with increase in “unknown/other” between 1980 to 1999 and 2000 to 2019 is also reflective of the shift to Hispanic/Latino as this ethnicity was reported as white or other/unknown more often in 2000 to 2019. This shift in ethnicity may be due to several factors, all involving the emergence of *Rh*. *sanguineus* as an important tick vector for RMSF in the southwestern US and Mexico. There are several recent reports of *Rh*. *sanguineus* s.l. detections and infestations in southern and central California [29, 35, 46–49]. There is evidence that populations of *Rh*. *sanguineus* s.l. in Southern California represent a recent expansion of the tropical lineage from Mexico, coexisting with the temperate lineage found in other parts of California and in Arizona [49, 50]. Infection of *Rh*. *sanguineus* ticks with *R*. *rickettsii* in these counties is rare and when found, is at a low prevalence (less than 2.8%) [35, 36]. Other SFGR, including *R*. *belli* [29] and *R*. *massiliae* [48], are also detected in *Rh*. *sanguineus* ticks from these areas. Although *R*. *belli* has never been implicated with human illness, *R*. *massiliae* has been documented as a pathogen of humans in Europe and South America [51]. Case 2 and recent epidemiological data support that exposure to a RMSF-hyperendemic area such Mexicali, Mexico, or living in areas where *Rh*. *sanguineus* populations are emerging, may increase RMSF exposure risk for people and their pets [36, 37,38]. Health disparities associated with rickettsial infections have been suggested [52] due to underserved populations’ inability to access health care in a timely fashion. In California, Hispanic and Latino populations are considered subject to health disparities for a number of factors [53]; thus the increase in RMSF incidence detected in this population may be greater than reported.

Like much of the US, reports of RMSF in California are increasing, particularly the number of probable cases. The inclusion of RMSF in the more general SFGR surveillance in 2010 has likely contributed to the increase in probable cases. We used a strict RMSF case definition to specifically describe RMSF epidemiology in California, and did exclude three confirmed SFRG cases that presented with eschars as the principal clinical presentation since clinically those would be more typical of Pacific Coast Tick fever caused by *Rickettsia* 364D [40] and excluded 33 probable cases with titers <1:164 that did not increase or had IgM only testing. As a result, the number of California’s reported confirmed and probable cases was less than what was reported to CDC for this study’s time period. Nonetheless, from 1980 to 2000, the incidence of RMSF in our study population significantly rose 1.6 times from 0.04 cases per million to 0.07 cases per million.

Similar to the nationwide trend, while the number of confirmed cases reported each year in California has remained fairly stable at zero to three cases each year, the percentage of reported cases that are confirmed has decreased [11, 12]. This pattern has been ascribed to clinical diagnosis and treatment that is based on one titer [12], or potential of previous infection with cross reactive *Rickettsia* spp. [54]. In California, though no other *Rickettsia* species besides *R*. *rickettsii* and *Rickettsia 364D* have been detected from humans, other *Rickettsia* spp. that may cross-react serologically with *R*. *rickettsii* include *R*. *massilae*, also found in *Rh*. *sanguineus* s.l. and implicated in canine illness [48, 55], and *R*. *belli* and *R*. *rhipicephali* found in *Dermacentor* spp. ticks [19, 22, 29, 30, 40]. Interestingly, 22% of all probable California cases had a second serum specimen obtained with a resultant titer that did not exceed the initial specimen, i.e., did not demonstrate a seroconversion. In this context, these stationary titers could represent the initial serology being collected after the acute phase, existing *Rickettsia* antibody titers caused by previous exposure to *R*. *rickettsii*, or exposures to other *Rickettsia* species, as has been described in other regions of the US [16]. In this study, most confirmed cases (87%) were identified by a four-fold or greater change in titer. Given the latency of antibody development (at least one week) [4], confirmation of cases with serology can be challenging. In the optimum scenario, appropriate treatment may be initiated prior to titer development which may abrogate the titer rise [7], or in the event of clinical improvement, there is little motivation to collect a second titer. In the worst-case scenario, doxycycline is not administered, and the patient may succumb before specific diagnosis is made [7] as occurred in Case 1. Molecular tests such as species-specific real-time PCR of rash biopsies can provide more specific and rapid results for confirmation [56] and have been shown to be a valuable tool for identifying specific *Rickettsia* spp. such as *Rickettsia* 364D from eschars of Pacific Coast tick fever in California [13, 40]. However, these diagnostic tests are currently limited in availability through CDPH, CDC, and some public health laboratories [12].

Onset of disease was reported in all months of the year with a marked peak in June. The peak remained consistent during the 40-year period of surveillance and was not influenced by in-state versus out-of-state exposure. This is typical for current RMSF seasonality in the US [57], but is a slight shift from the first description of RMSF in California when the peak was in May [28]. All three implicated tick vector species in California have seasonality patterns that could contribute to a June peak. Adult *D*. *occidentalis* seasonality has been characterized in various habitats as November through June with a peak in spring; the nymphal stage is most active from May through September [27, 28, 40]. *Dermacentor similis* seasonality is less well characterized with adult ticks reported from January through October with a peak in May or June [58]; the nymphal stage is most active in early summer [27, 28]. *Rhipicephalus sanguineus* s.l. are prevalent and reproduce throughout the year [28, 40], potentially exposing people and dogs to all life stages simultaneously and may explain the onset dates during the winter reported among RMSF cases 2000 to 2019. However, aggressive host-seeking and increased predilection to bite humans have been suggested to intensify in this tick species with rising temperatures [59], consistent with the June peak in human cases of RMSF. The June peak, present over both time periods, likely also reflects human activity in tick habitat [58] and is typical of peaks of other tick-borne disease incidence in California such as Lyme disease [60].

Among RMSF cases reported from 2011 to 2019 for which detailed symptoms were available, the nonspecific symptoms of fever, headache, chills, and rash were most common and reported in proportions similar to other case summaries [1, 4]. In one study comparing RMSF and other nonspecific illnesses, the inclusion of tick exposure along with fever and rash was a diagnostic combination slightly more common in the RMSF cases [61], underscoring the importance of assessing vector exposure in the diagnosis of these nonspecific illnesses. In our study, case-patients, particularly confirmed cases, could often recall an insect bite, and typically identify it as a tick bite.

Of note, the clinical severity of the cases seems to be greater in terms of hospitalization [67% for all cases and 57% for electronically reported cases) compared to the national average of 23% [11]. Similarly, the case fatality (6% over the last 20 years) is higher than nationally reported rates of spotted fever rickettsiosis over the same period that ranged from 1% to 3.4% [5, 7, 11]. While the slightly higher hospitalization and case fatality may be due in part to including those cases that meet a more stringent inclusion criteria than other studies, it is still concerning that the three fatal cases occurred in 2012 and 2014 despite publications and public health campaigns designed to increase physician awareness about the use of doxycycline with suspected RMSF [2, 4, 62]. This underscores the challenge in recognizing RMSF, particularly among case-patients from urban areas where the potential for tick exposure may not be considered and patients do not report insect bite exposure.

The passive nature of data collection in this study is a limitation, particularly in assessing clinical presentation based on a preset list of symptoms, and the potential of under-reporting of true cases due to limitations of serologic testing [7]. Conversely, the nonspecific nature of the serologic testing may also contribute to reporting of cases that are not RMSF, e.g., other SFGR or sero-reactive cases due to prior infection with other *Rickettsia* species or reporting of low positive titers. We attempted to address the latter bias by limiting the probable cases to those with reciprocal IgG titers ≥ 128 to increase specificity, recognizing that some cases may be more accurately described as SFGR.

## Conclusion

Similar to other areas of the southwest United States and in Mexico [32, 63], RMSF is a disease of concern in California, particularly with the established presence of two ecological cycles (one in *Dermacentor* spp. ticks and one in *Rh*. *sanguineus* ticks) and increase in exposure to *Rh*. *sanguineus* s.l. ticks [49]. SFGR disease is reported as RMSF or SFGR (excluding RMSF) in California, though differentiation of RMSF and SFGR disease caused by other *Rickettsia* spp. is a challenge, given the established presence of zoonotic *Rickettsia* 364D, detection of other *Rickettsia* spp. in California ticks, and limitations of serology to speciate the pathogen. To date, only *R*. *rickettsii* and *Rickettsia* 364D have been molecularly characterized from humans though future molecular advances may identify other zoonotic *Rickettsia*. Clinical awareness for the need of rapid appropriate treatment of RMSF is critical, particularly given the high hospitalization proportion seen in California cases. Clinical and epidemiologic data show that medical education materials should consider the sylvatic and peridomestic RMFS tick cycles and include a reminder to inquire about tick exposure even in an urban setting. Public health education material should be culturally appropriate for the populations at risk.

## Supporting information

S1 Data The Excel tables in the supplementary information contain aggregated data used to make Figs 1–5. Each figure is under a separate tab.
(XLSX)
