# Correction: A forty-year review of Rocky Mountain spotted fever cases in California shows clinical and epidemiologic changes

**DOI:** 10.1371/journal.pntd.0011030

**Published:** 2023-01-04

**Authors:** Anne M. Kjemtrup, Kerry Padgett, Christopher D. Paddock, Sharon Messenger, Jill K. Hacker, Tina Feiszli, Michael Melgar, Marco E. Metzger, Renjie Hu, Vicki L. Kramer

There is an error in the second sentence of the third paragraph of the Discussion section. It says, “We used a strict RMSF case definition to specifically describe RMSF epidemiology in California, and did exclude three confirmed SFRG cases that presented with eschars as the principal clinical presentation since clinically those would be more typical of Pacific Coast Tick fever caused by Rickettsia 364D [40] and excluded 33 probable cases with titers <1:164 that did not increase or had IgM only testing.” The titer value for exclusion should not be <1:164. It should be <1:64.
